# 

**Published:** 1903-02-28

**Authors:** 


### NOTICE TO CORRESPONDENTS.

All MSS., Letters, Books for Review, and other matters intended for the Editor should be addressed The Editor, "The Hospital," The Hospital Building, 28 & 29 Southampton Street, Strand, W.C.

The Editor cannot undertake to return rejected MS., even when accompanied by stamped directed envelope.

**Notice to Advertisers and Subscribers.**

All Advertisements, Orders for Copies of The Hospital, and Business Communications should be addressed to **THE MANAGER, (Not to the Editor)**, The Hospital, 28 & 29 Southampton Street, Strand, London, W.C.

The Cost of Subscription, post free, is as follows.—Per week, 2½d.; per quarter, 2s. 8d.; per half-year, 5s. 3d.; per annum, 10s. 6d. Foreign:—Per annum, 15s. 2d., payable, in all cases, in advance.

**NOTICE.—Vol. XXXII. of "The Hospital" (April to September 1902) in handsome cloth binding is now on sale at the office of this Journal, price, 7s. 6d. Cases for binding the Numbers contained in this Volume 1s. 6d. Index to Vol. XXXII., 2½d., post free.**

**The Monthly Part for March will be ready on March 2. Price 6d., by post 8d.**

### BOOKS RECEIVED.

BAILLIÈRE, TINDALL AND COX.

"Refraction of the Eye." By Ernest Clarke.

"Aids to Sanitary Science." By Allan and Farrar.

"Practical Details of Cataract Expansion." By H. Herbert

"Cancer, its Causation and Curability without Operation." By Robert Bell.

"A Manual of Toxicology." By A. H. Brundage, M.D.

REBMAN, LIMITED.

"Biographic Clinics." By G. M. Gould, M.D.

"Medical and Surgical Reports of the Boston City Hospital, 1902."

J. B. LIPPINCOTT CO.

"International Clinics." Vol. IV. Twelfth Series.

"A Treatise of Massage." By — Graham.

"Lippincott's Pocket Medical Dictionary."

"Genito-urinary and Venereal Diseases." By White and Martin. Fifth Edition.

THE SCIENTIFIC PRESS, LIMITED.

"The Nurse in Hot Climates." By Dr. E. Henderson.

"Handbook for Nurses." By Dr. J. K. Watson. New and Revised Edition.

"The Midwives' Pocket Book." By Miss Honnor Morten.

CASSELL AND COMPANY, LIMITED.

"Diseases of Women." By George E. Herman, M.D.

W. H. WHITE AND SON, EDINBURGH.

"The Gates of Afree, a Romance of the New Empire." By H. Van Laun.

DR. W. R. WILLIAMS.

"Cancer. Theories of the Parasitic Organ."

J. AND A. CHURCHILL.

"Lunacy and Law." By Sir Wm. R. Gowers, M.D.

HADDEN, BEST AND CO.

"An Analysis of the Education Act, 1902." By H. B. N. Mothersole.

H. K. LEWIS.

"Massage and the Swedish Movements." By Ostrom. Fifth Edition.

SIMPKIN, MARSHALL AND CO.

"Education, Disciplinary, Civic, and Moral." By L. W. Williams.

### The Student of Medicine.

### APPOINTMENTS.

**William Anderson**, M.A., M.B., B.Ch.Aberd., appointed Second House Surgeon to the Royal London Ophthalmic Hospital, City Road, E.C. **H. G. Berry**, M.R.C.S., L.R.C.P.Lond., appointed District Medical Officer of the Aylsham Union. **A. F. Blake**, M.R.C.S., L R.C.P.Lond., appointed Medical Officer for the Grange District of the Orsett Union. **W. F. Brown**, M.B., M.S.Glasg., appointed Medical Officer of Health and Police Surgeon for the Burgh of Ayr. **Frank Bryan**, B.C.Cantab., appointed House Physician to the Derbyshire Royal Infirmary. **Colin A. Campbell**, M.D.Toronto, appointed Third House Surgeon to the Royal London Ophthalmic Hospital, City Road, E.C. **Major P. Carr-White**, M.B., Indian Medical Service, appointed Clinical Assistant to the Samaritan Free Hospital for Women. **Lucy B. Clapham**, M.B.Lond., appointed Assistant Anæsthetist to the New Hospital for Women, London. **John D'Ewart**, M.R.C.S., L.R.C.P., appointed Third Resident Medical Officer Crumpsall Workhouse. **George Dick**, M.B., Ch.B.Edin., appointed Medical Officer of Health for the Counties of Sutherland and Caithness. **H. A. Duffett**, M.R.C.S.Eng., L.R.C.P.Lond., appointed Medical Officer to the School Homes of the Greenwich Union. **C. M. Hope**, M.B., Ch.B.Glasg., appointed House Surgeon to the Derbyshire Royal Infirmary. **William S. Inman**, M.B.Lond., L.S.A., appointed Senior House Surgeon to the Royal London Ophthalmic Hospital, City Road, E.C. **J. H. Lyell**, M.D.Glasg., C.M., appointed Outdoor Surgeon to the Perth Royal Infirmary. **C. E. Maguire**, M.D.Aberd., appointed a Government Medical Officer, Fiji, on transfer from Southern Nigeria. **R. P. Marshall**, M.R.C.S Eng., L.R.C.P.Lond., appointed District Medical Officer of the St. Olave's Union. **A. A. Myers**, L.R.C.P., M.R.C.S.Eng., appointed Assistant House Surgeon to the Derbyshire Royal Infirmary. **H. J. Palmer**, L.R.C.P., L.R.C.S.Edin., appointed District Medical Officer of the Caxton and Arrington Union. **E. L. Paton**, M.A., M.B., C.M.Glasg., appointed Visiting Surgeon to the Perth Royal Infirmary. **Robert Stirling**, M.A., M.D., F.R.C.S.Edin., reappointed Visiting Surgeon to the Perth Royal Infirmary. **W. Taylor**, M.B., C.M.Edin., appointed Visiting Surgeon to the Perth Royal Infirmary. **Tom Walker**, M.R.C.S., L.R.C.P., appointed Second Resident Assistant Medical Officer Crumpsall Workhouse. **Ernest Wethered**, M.B.Lond., L.R.C.P., M.R.C.S.Eng., appointed House Surgeon to the Derbyshire Royal Infirmary.

### Useful Books for Nurses' Libraries.

### NURSING.

### A HANDBOOK FOR NURSES.

By J. K. Watson, M.D., M.B., C.M.
New and Revised Edition.
Crown 8vo., profusely illustrated, cloth gilt.
5/- net, 5/4 post free.

### NURSING: ITS THEORY AND PRACTICE.

Being a complete Text-book of Medical, Surgical, and Monthly Nursing.
By Percy C. Lewis, M.D., M.R.C.S., L.S.A.
New Edition, Enlarged and Revised (17th thousand), profusely illustrated with over 100 new Cuts. Crown 8vo., cloth gilt.
3/6 post free.

### MIDWIFERY.

### A PRACTICAL HANDBOOK OF MIDWIFERY.

By Francis W. Haultain, M.D.
Now Ready.
New and Revised Edition.
Profusely illustrated with Original Cuts, Tables, &c.
Handsomely bound in leather.
6/- net, 6/3 post free.

### MIDWIVES' POCKET BOOK.

(With chapter on the Midwives Bill).
By Honnor Morten.
Small crown 8vo., cloth.
1/- post free.
An exceedingly useful handbook for those entering for the L.O.S. certificate as well as for qualified Midwives.

### INSANITY.

### OUTLINES OF INSANITY.

A Popular Treatise on the Salient Features of Insanity.
By Francis H. Walmsley, M.D.
Demy 8vo., cloth extra.
3/6 post free.
Popular Edition, stitched.
1/6 post free.

### MENTAL NURSING.

By William Harding, M.D. Ed.; M.R.C.P. Lond.
New and Popular Edition, small crown 8vo., cloth.
1/- post free.

### MASSAGE AND MECHANICAL THERAPEUTICS.

### ART OF MASSAGE.

By Mrs. Creighton Hale.
Second Edition.
Demy 8vo., 150 pp., profusely illustrated with over 70 Special Cuts. Cloth gilt.
6/- post free.

### MEDICAL GYMNASTICS, INCLUDING THE SCHOTT (NAUHEIM) MOVEMENTS.

Being a Text-book of Massage and Mechanical Therapeutics Generally, for Medical Students and others.
By Axel V. Grafstrom, B.Sc., M.D.
Crown 8vo., Illustrated, cloth.
2/6 post free.

### SURGICAL WARD-WORK.

### SURGICAL WARD-WORK AND NURSING.

By Alexander Miles, M.D.Edin., C.M., F.R.C.S.E.
Second Edition, Enlarged and entirely Revised.
Demy 8vo., copiously illustrated, cloth gilt.
3/6 net, 3/10 post free.

*COMPLETE CATALOGUE POST FREE ON APPLICATION.*

*Of all Booksellers, or of*

THE SCIENTIFIC PRESS, LTD., 28 & 29 Southampton Street, Strand, London, W.C.

